# Exploratory Decision-Making as a Function of Lifelong Experience, Not Cognitive Decline

**DOI:** 10.1037/xge0000133

**Published:** 2016-01-04

**Authors:** Nathaniel J. Blanco, Bradley C. Love, Michael Ramscar, A. Ross Otto, Kirsten Smayda, W. Todd Maddox

**Affiliations:** 1 Department of Psychology, University of Texas, Austin; 2 Department of Experimental Psychology, University College London; 3 Department of Linguistics, University of Tübingen; 4 Center for Neural Science, New York University; 5 Department of Psychology, University of Texas, Austin

**Keywords:** decision-making, frontostriatal striatum, aging, exploration

## Abstract

Older adults perform worse than younger adults in some complex decision-making scenarios, which is commonly attributed to age-related declines in striatal and frontostriatal processing. Recently, this popular account has been challenged by work that considered how older adults’ performance may differ as a function of greater knowledge and experience, and by work showing that, in some cases, older adults outperform younger adults in complex decision-making tasks. In light of this controversy, we examined the performance of older and younger adults in an exploratory choice task that is amenable to model-based analyses and ostensibly not reliant on prior knowledge. Exploration is a critical aspect of decision-making poorly understood across the life span. Across 2 experiments, we addressed (a) how older and younger adults differ in exploratory choice and (b) to what extent observed differences reflect processing capacity declines. Model-based analyses suggested that the strategies used by the 2 groups were qualitatively different, resulting in relatively worse performance for older adults in 1 decision-making environment but equal performance in another. Little evidence was found that differences in processing capacity drove performance differences. Rather the results suggested that older adults’ performance might result from applying a strategy that may have been shaped by their wealth of real-word decision-making experience. While this strategy is likely to be effective in the real world, it is ill suited to some decision environments. These results underscore the importance of taking into account effects of experience in aging studies, even for tasks that do not obviously tap past experiences.

As adults age, some will occupy positions of responsibility in which they make decisions that affect government, business, and finance; most will face complex choices related to health and retirement planning. And, as is highlighted by the complex issues concerning the disposal of their worldly assets (Peisah et al., 2009), many older adults will be incapacitated by diseases that lead to them losing their legal right to make choices. Understanding the cognitive and neural basis of age-related changes in decision-making, along with distinguishing healthy age-related changes from neurodegenerative disease changes, represent important questions for cognitive aging research.

Adult performance on various measures of cognition—such as those testing reasoning, memory, and response speeds—changes with normal aging (Salthouse, 2009). Decision-making is a particularly important domain in which age-related behavioral differences clearly manifest themselves and where understanding their causes may be of considerable consequence (Chowdhury et al., 2013; Denburg, Tranel, & Bechara, 2005; Samanez-Larkin, Kuhnen, Yoo, & Knutson, 2010). Recent work has begun to focus on decision-making in scenarios with a complex underlying task structure, where goal-directed processes using a rich task representation become necessary for optimal performance (Cooper, Worthy, Gorlick, & Maddox, 2013; Eppinger, Walter, Heekeren, & Li, 2013; Worthy, Cooper, Byrne, Gorlick, & Maddox, 2014; Worthy, Gorlick, Pacheco, Schnyer, & Maddox, 2011; Worthy & Maddox, 2012). The results of these studies have been mixed: in some cases, older adults perform better than younger adults at this type of decision-making (Cooper et al., 2013; Worthy et al., 2011; Worthy et al., 2014; Worthy & Maddox, 2012); in other studies, they do worse than younger adults (Eppinger, Walter, et al., 2013). As yet, the reasons for these contradictory results are poorly understood.

Age-related cognitive changes have traditionally been interpreted as revealing that these processes decline across the life span (Burke & Barnes, 2006; Head, Kennedy, Rodrigue, & Raz, 2009; Salthouse, 2004). For example, several studies have found reduced striatal and frontostriatal representations of reward prediction errors—a critical component of learning and decision-making—in older adults (Eppinger, Schuck, Nystrom, & Cohen, 2013; Samanez-Larkin, Worthy, Mata, McClure, & Knutson, 2014) and interpreted these findings as evidence of a decline in the normal dopamine function in the striatum. In a similar vein, these changes are often explained in terms of compromised white matter integrity in frontostriatal circuits (Samanez-Larkin, Levens, Perry, Dougherty, & Knutson, 2012).

However, it has recently been argued that changes in performance on processing tests are difficult to interpret in the absence of models of processes, and without controlling for the confounds introduced by the extra knowledge that older adults can reasonably be expected to have acquired (Li, Baldassi, Johnson, & Weber, 2013; Ramscar, Hendrix, Shaoul, Milin, & Baayen, 2014). Indeed, in domains such as response speeds in lexical decision-making and paired-associate learning, it has been shown that observed age-related performance changes are consistent with the predictions of standard processing models when *knowledge effects*—the extra processing loads that can be expected as knowledge increases with experience—are controlled for (Ramscar, Hendrix, Love, & Baayen, 2013; Ramscar et al., 2014). As an example, suppose a graphic designer and another person were asked to name the color of an object. The graphic designer may know many different color names, and so may take longer to search for a response than the other person who simply and quickly responds “red.” The greater amount of relevant knowledge may reduce the graphic designer’s speed, but that does not mean she is impaired at color naming.

Accounts invoking capacity limitations due to cognitive decline are common explanations of age-related decision-making changes, but knowledge effects are rarely considered. That older adults sometimes outperform younger adults in complex decision-making tasks highlights the importance of considering the effects of experience and knowledge, because capacity limitations cannot obviously account for these results. Knowledge effects likely also come into play where older adults perform worse than younger adults, particularly when the task violates expectations that are based on prior experience.

In what follows, we examined the performance of older and younger adults in an exploratory choice task. Exploration—actively seeking more information in the face of choice uncertainty—is a crucial aspect of many kinds of decision processes and yet has been little investigated in the domain of aging.

## Exploratory Decision-Making

Effective decision-making requires an appropriate balance of exploration and exploitation, as well as some understanding of when one is more beneficial than the other. It is important to get both the amount and the timing of exploratory choices right in order to explore optimally. To put it another way, we can evaluate exploratory decision-making strategies in terms of both quantity and quality. While the amount of exploration is important for effective decision-making, the timing of exploration is equally if not more important. Ideally, exploratory choices should be directed when and where there is more uncertainty in the environment. As things change over time, the current state of parts of the environment that have not been checked recently become more and more uncertain. The most information is gained from exploring when uncertainty is highest.

### Reflective Versus Reflexive Strategies

To formally study these important aspects of decision-making in the lab, we employed the *leapfrog task* (Knox, Otto, Stone, & Love, 2012). This task has proven effective in distinguishing the use of various strategies in exploratory decision-making (Blanco, Otto, Maddox, Beevers, & Love, 2013; Blanco et al., 2015), and it does not require or obviously invoke specific aspects of participants’ prior knowledge. In the task (which is described in more detail below in the Procedure section), participants choose between two options and receive a reward. During the task, the rewards received for each of the two options leapfrog over one another, alternating in superiority. This means that it is not clear on any given trial whether an option that was previously lower in value has increased, surpassing the option that was previously more rewarding. Accordingly, exploration is necessary to determine the currently higher option.

We distinguish two types of learning and decision-making that differ crucially in how they influence the timing of exploratory choices in this task: *reflexive* and *reflective* decision-making. *Reflexive decision-making* (which leads to “random” exploration) is thought to be more habitual or automatic, computationally cheap, and is analogous to “model-free” learning (Daw, Niv, & Dayan, 2005). Within the leapfrog task, reflexive participants will tend to exploit the choice last observed to be most rewarding, and the interspersed exploration choices will be unpredictable.

*Reflective decision-making* is more goal-directed, relatively computationally expensive, depends on executive processing (Badre, Doll, Long, & Frank, 2012; Blanco et al., 2013; Otto, Knox, Markman, & Love, 2014), and is analogous to “model-based” learning. Reflective learning involves building a rich mental representation of the environment. This representation can allow the learner to use uncertainty in the environment to direct exploratory choices when they will be more effective (Knox et al., 2012). Maintaining the rich representation that enables tracking uncertainty involves using greater cognitive processing resources. Reflective decision-making results in greater cognitive demand than reflexive decision-making.

Otto et al. (2014) showed that when executive resources are compromised by adding a secondary task to the leapfrog paradigm, reflective strategy use dropped substantially, providing evidence that reflective processing is dependent on executive processing. In summary, reflective decision-making is resource-dependent and involves drawing on knowledge of the task environment. Available cognitive resources may vary across the life span, which could affect the ability of older adults to engage in reflective processing.

**Figure 1 fig1:**
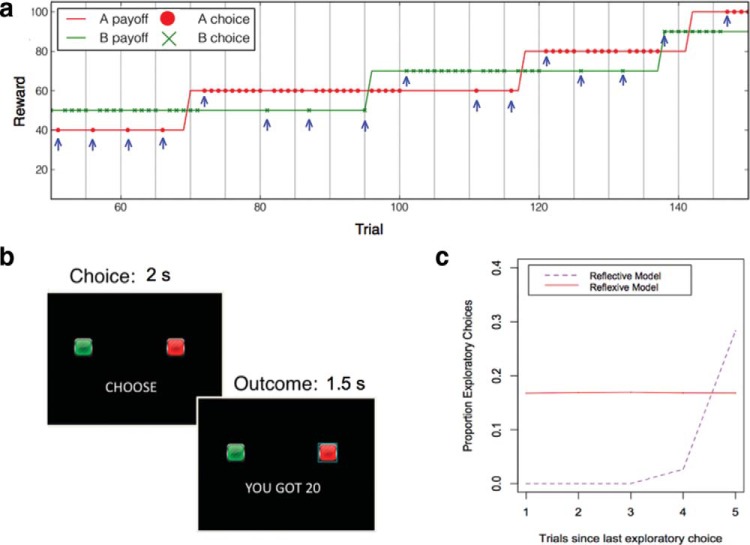
The Leapfrog task. A sample participant’s choices over 100 trials (a). With a fixed probability of 0.075 per trial, the lower option can jump in reward by 20 points, surpassing the other option. A participant must explore to discover that the jump has taken place. The two options “leapfrog” over each other, alternating as the currently superior option. Lines represent the true reward values; dots represent a participant’s choices. Blue arrows point out exploratory choices. The temporal dynamics of a single trial (b). Participants are given 2 s in which to make a choice, after which the points received for the choice is displayed for 1.5 s. The patterns of choice characteristic of reflexive and good reflective choice strategies in this task, produced by simulating the ideal reflexive and reflective models of the task (see Appendix A) (c). See the online article for the color version of this figure.

Reflective and reflexive decision-making strategies make clearly divergent predictions about the sequential structure of choices in the leapfrog task (see Figure 1c). To demonstrate this difference, we looked at a measure we refer to as the *hazard rate* of exploration, which is the exploration rate as a function of the number of trials since the last time an exploratory choice was made. Reflexive actors do not represent the dynamics of the environment and so cannot base exploratory decisions on uncertainty, resulting in no sequential structure in exploratory choices. A reflexive strategy leads to an equal probability of exploring on every trial, producing a flat pattern of hazard rates.[Fig-anchor fig1]

Reflective actors, on the other hand, lead to patterns that are not flat. While any deviation from a flat pattern indicates some kind of reflective strategy, a monotonically increasing pattern in the hazard rate of exploratory choices is the hallmark of a good reflective strategy for the standard leapfrog task (see Figure 1c). As one continually exploits in this task, the probability that an unobserved jump in reward values has occurred increases, which makes the current state of the environment less certain and exploration more valuable. A reflective actor can use this uncertainty to guide its choices.

### Knowledge Effects

Like many tasks used to study decision-making, the leapfrog task is typically thought not to involve prior knowledge, but, in light of the recent studies demonstrating knowledge effects, we consider in our experiments ways in which prior experience and knowledge may influence participants’ behavior in this task.

Effectively tracking and using uncertainty requires both the cognitive capacity to reflectively track probabilities over time and an accurate mental representation of the task. Lacking either one may impair decision-making performance. It is possible to have adequate cognitive processing capacity but underperform due to having the wrong internal model of the task environment. And so, when people perform suboptimally on decision-making tasks, it is especially important to consider *how* they deviate from the optimal strategy and what type of strategy they may be using.

It is likely that increased knowledge or experience influences the environment representations that people tend to form, perhaps by biasing their expectations toward environment structures that occur frequently in daily life. In the standard version of the leapfrog task, the outcome of a trial (i.e., whether the lower option jumped up in reward) is sequentially independent from other trials. The probability that a jump has occurred depends only on the base rate of a jump happening. But the sequentially independent nature of the task may be difficult for participants to infer. In many scenarios, humans tend to be poor stochastic reasoners, and appear to be biased to see the effects of positive or negative autocorrelation in sequences (i.e., the *hot hand* and the *gambler’s* fallacies; Falk & Konold, 1997; Scheibehenne, Wilke, & Todd, 2011) even when choice points are objectively independent, such as in the tossing of a fair coin (indeed, other primates seem prone to the same bias; Blanchard, Wilke, & Hayden, 2014). Interestingly, the tendency to reason in this way seems to be influenced by experience (Barron & Leider, 2010), and older adults may be more likely to engage in this type of reasoning (Castel, Rossi, & McGillivray, 2012).

An important point to consider is that whether it is fallacious to base reasoning on perceived positive or negative autocorrelation in sequences ultimately depends on the actual structure of the environment (Alloy & Tabachnik, 1984; Fawcett et al., 2014). In fact, we speculate that when estimating probabilities in the real world, the gambler’s fallacy is often an effective assumption. Behavior consistent with the gambler’s fallacy is reasonable in many scenarios where individual events are not sequentially independent, where the probability of a change does increase with time. For example, if you are checking to see if your milk has gone bad, even if it was not yesterday, you would probably be right in concluding that it is more likely to be bad today if you bought it 2 weeks ago as opposed to 2 days ago. Similarly, it can be effective in predicting outcomes in any system with a cyclical nature: If you are waiting at a stop light and look up to see whether the light is still red, the longer you wait increases the likelihood the light will turn green, regardless of how recently you last checked it. Or, if you start flipping through a deck of cards and the first 10 cards are all black, you would be correct to believe that the next card is more likely to be red than black. The probability of drawing a red card continually increases with each consecutive black card that you draw. The same type of reasoning is what makes card counting an effective strategy in blackjack. In these scenarios, we can say that the environment has “memory” in that the probability of each outcome depends on the previous outcomes. In contrast, if you shuffle each card back into the deck before drawing the next one (i.e., sample with replacement), the environment is “memory-less” and each sample is independent.

In most real-world tasks, events are rarely sequentially independent, and sampling likelihoods often favor patterns that reinforce apparent negative or positive correlations in sequences of events (Hahn & Warren, 2009, 2010). Accordingly, it seems likely that increased experience may bias this kind of strategic thinking.

### Goals and Predictions

Our goal in this work was twofold: First, we examined whether the performance of older and younger adults differed in this type of exploratory decision-making, and, if so, we wanted to characterize the nature of these changes. In Experiment 1, older and younger adults completed the standard, sequentially independent, version of the leapfrog task. Capacity limitations and a knowledge effect both predict that older adults should perform worse than younger adults at this task, but they make different predictions as to the types of strategies that older adults will use. If limited cognitive processing capacity impairs performance for older adults, we would expect them to use reflexive strategies more often than younger adults. If increased experience or knowledge influences performance, older adults may still tend to use reflective strategies, but may adopt a strategy that is not optimal for the environment (though would, presumably, be a well-learned strategy often effective in real decision environments). Specifically, we predicted that older adults would expect autocorrelation in the trial outcomes (as in the gambler’s and the hot hand fallacies), even though it is not there, and would make suboptimal choices based on this belief. These possibilities are not mutually exclusive, and both effects may be at work.

In Experiment 1, we found that older and younger adults differed in task performance, but not in the rate of exploratory choices. Further analyses suggested that qualitative strategy differences at least partially explain the performance differences. Experiment 2 investigated further by manipulating the underlying structure of the decision environment. Whether older adults exhibited performance deficits compared with younger adults depended crucially on the environment, supporting a qualitative strategy shift rather than a capacity limitation explanation of the age differences in this task. We speculated that these differences would arise from increased experience with and knowledge of decision-making in the real world.

Finally, we hoped to offer a more complete characterization of the cognitive basis of the changes in decision-making across the life span by comparing our findings and analyses with others in the literature, and discussing the importance of modeling change (Hills, Mata, Wilke, & Samanez-Larkin, 2013; Mata & Nunes, 2010)—and not merely measuring it—in our understanding of lifelong development in cognitive and neural processes.

## Experiment 1

### Method

#### Participants

Participants were recruited from the general Austin, Texas, community and were paid $10 per hour for participation. Participants were 58 younger adults (age range: 17−32 years) and 52 older adults (age range: 60−88 years). The mean age of the older adult sample was 67.02 years; the mean age of the younger adult sample was 21.81 years. The lower bound of 60 years was chosen for our older adult sample to be consistent with previous work (e.g., Cooper et al., 2013; Worthy et al., 2014; Worthy & Maddox, 2012). The older adult sample was 59.62% female; the younger adult sample was 60.34% female.

#### Neuropsychological testing

Prior to the main experimental session, older adults were given a series of standardized neuropsychological tests to assess general cognitive ability across attention (Wechsler Adult Intelligence Scale, Third Edition [WAIS-III], Digit Span; Wechsler, 1997), executive functioning (Trail-Making Test [TMT] Parts A and B; Lezak, 1995; FAS and Wisconsin Card Sorting Task [WCST]; Heaton, 1981), and memor*y* (California Verbal Learning Test [CVLT]; Fridlund & Delis, 1987). These tests served as a screen to ensure that our older adult sample included healthy, high functioning individuals.

Table 1 shows the means, standard deviations, and ranges of standardized *z* scores on each test for the older adults. Normative scores for each participant were calculated for each neuropsychological test using standard age-appropriate published norms. All WAIS subtest percentiles were calculated according to the testing instructions and then converted to standardized *z* scores. The CVLT and WCST standardized *T* scores were calculated according to testing directions and converted to *z* scores. TMT standard *z* scores were calculated according to the testing instructions. Only participants who were within normative ranges were invited to participate in the main experiment. Exclusion criteria included scoring more than 2 *SD* below the standardized mean on more than one neuropsychological test in the same area (memory, executive functioning, or attention), though no participants were excluded from participation based on these criteria.[Table-anchor tbl1]

#### Procedure

Each participant completed 200 trials of the leapfrog task (see Figure 1a). On each trial, participants choose one of two options and receive a reward. At the beginning of the experiment, one option is worth 10 points and the other 20 points. As the task progresses, on any trial, the previously lower option could permanently increase in its reward value by 20 points, such that its value would surpass that of the other option. This happened with a fixed random probability (*p* = .075) per trial.

Over the course of the experiment, the reward values received for each of the two options leapfrog over one another, which means that the choice that is the superior option on any given trial changes (see Figure 1a). On each trial, one option is always worth 10 more points than the other option, but exploration is necessary to determine which option is actually the higher one at any given point. The task thus effectively reduces participants’ choices on each trial to deciding whether to explore or exploit (Blanco et al., 2013; Knox et al., 2012). A participant can either choose the option with the highest seen reward (i.e., exploit), or explore to see whether the other option has jumped up in value.

On each trial, the word CHOOSE appeared on the screen, and participants were given 2 s to respond by choosing one of two options (see Figure 1b). They responded by pressing a designated key on the keyboard. The chosen option was highlighted for the remainder of the trial. The reward received for the choice (e.g., “+ 60”) was then presented in the center of the screen for 1.5 s. If the participant did not respond in time, a large red X was displayed in place of the reward along with the message “TOO SLOW, TRY AGAIN,” and the trial was repeated. Immediately following reward presentation, the next trial began. Participants were given a break after each block of 50 trials. Breaks lasted for 10 s, after which the experiment continued.

Prior to the main task, participants passively viewed 200 training trials to acclimate to the rate of change in reward values. Each training trial lasted 0.5 s, immediately followed by the next trial. The current trial number was displayed at the center of the screen at all times. Reward values for the options were not shown. Instead, an arrow indicated when a jump in rewards occurred by pointing to the option that changed. Before the second block of 100 training trials, participants were asked to estimate the number of jumps they expected to see in that block.

Participants were informed that both options would increase in points over the course of the experiment, that the two options would alternate at being the better option, and that the only way to know which one was currently better was to sample the options. They were instructed that their goal was to earn as many points as possible during the experiment.

### Results

#### Performance, reaction times, and exploration rates

**Figure 2 fig2:**
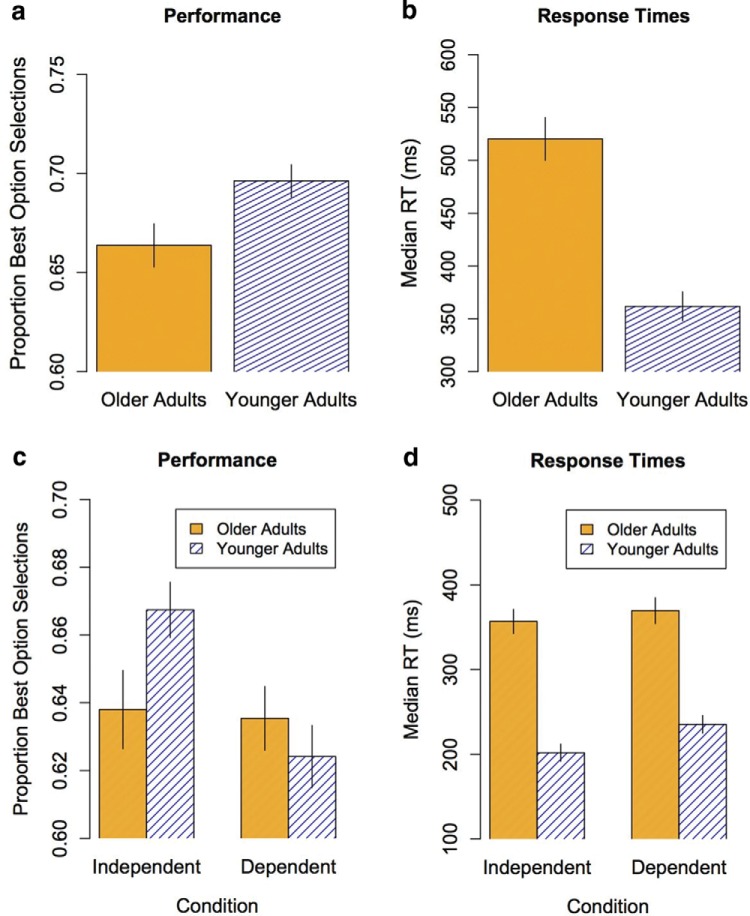
Performance and response times. Experiment 1 performance (a). Younger adults performed better than older adults. Experiment 1 response times (b). Younger adults responded more quickly than older adults. Experiment 2 performance (c). Younger adults perform better than older adults on the independent condition, replicating the results from Experiment 1, but performance did not differ between the groups on the dependent condition. Experiment 2 response times (d). Younger adults responded more quickly in both conditions. Error bars reflect standard errors. See the online article for the color version of this figure.

Performance on the leapfrog task was measured as the proportion of trials on which the participant chose the option that was truly higher on that trial. Overall, younger adults scored higher (0.696 vs. 0.664) than older adults, *t*(108) = 2.402, *p* = .018, *d* = 0.459 (see Figure 2a). We also found that younger adults responded more quickly (median reaction time [RT]: 432 ms vs. 593 ms) than older adults, as indicated by median RT, *t*(108) = 6.577, *p* < .001, *d* = 1.259 (see Figure 2b).[Fig-anchor fig2]

We defined an exploitive choice as choosing the option that had the highest observed reward prior to the current trial (see Figure 1), and thus selecting the other option (i.e., the option that was previously observed to be lower of value) would be considered an exploratory choice. Both older adults and younger adults explored on approximately 17% of trials (16.69% for older vs. 16.74% for younger adults), *t*(108) = 0.036, *p* = .971, *d* = 0.007, the remaining proportion of trials constituting exploitative choices. See Appendix B for discussion of the distributions of performance and exploration rate for both groups.

#### Reflective versus reflexive strategies

**Figure 3 fig3:**
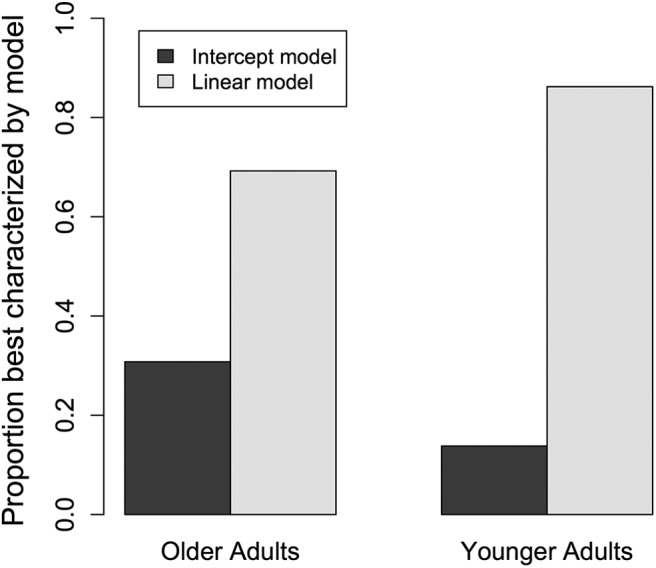
Regression modeling results (Experiment 1). Most participants in both groups were best characterized by the reflective linear model, which represents a good reflective strategy, though a greater proportion of younger adults were better characterized by the linear model than older adults.

For each participant, we applied two regression models to their hazard rates to determine whether their choices were more consistent with a reflexive or a good reflective approach (see Figure 1c). The first was a model that included only an intercept term (henceforth, the intercept model). This model predicts a flat pattern of hazard rates and corresponds to a reflexive strategy. The second model included both an intercept and a linear term (the linear model), predicting the probability of exploring as a linear function of the number of trials since the last exploratory choice. We constrained the linear model to have a linear term (i.e., slope) that was positive because a negative slope would not correspond to a good reflective strategy for this task. We compared the two regression models using the Bayesian information criterion (BIC; Schwarz, 1978) to find the model that best characterized each participant. This revealed that younger adults were more often best characterized by the reflective linear model than were older adults, χ^2^ = 5.576, *p* = .018, ϕ = 0.225, though most participants in both groups were best fit by this model (see Figure 3). Fifty of 58 younger adults were better characterized by the linear model compared with only 35 of 52 older adults. Mean BIC values for older adults were 146.9 (*SD*=115.0) for the intercept model and 134.3 (*SD*=112.6) for the linear model. Mean BIC values for younger adults were 166.6 (*SD*=62.3) for the intercept model and 144.1 (*SD*=57.5) for the linear model.[Fig-anchor fig3]

**Figure 4 fig4:**
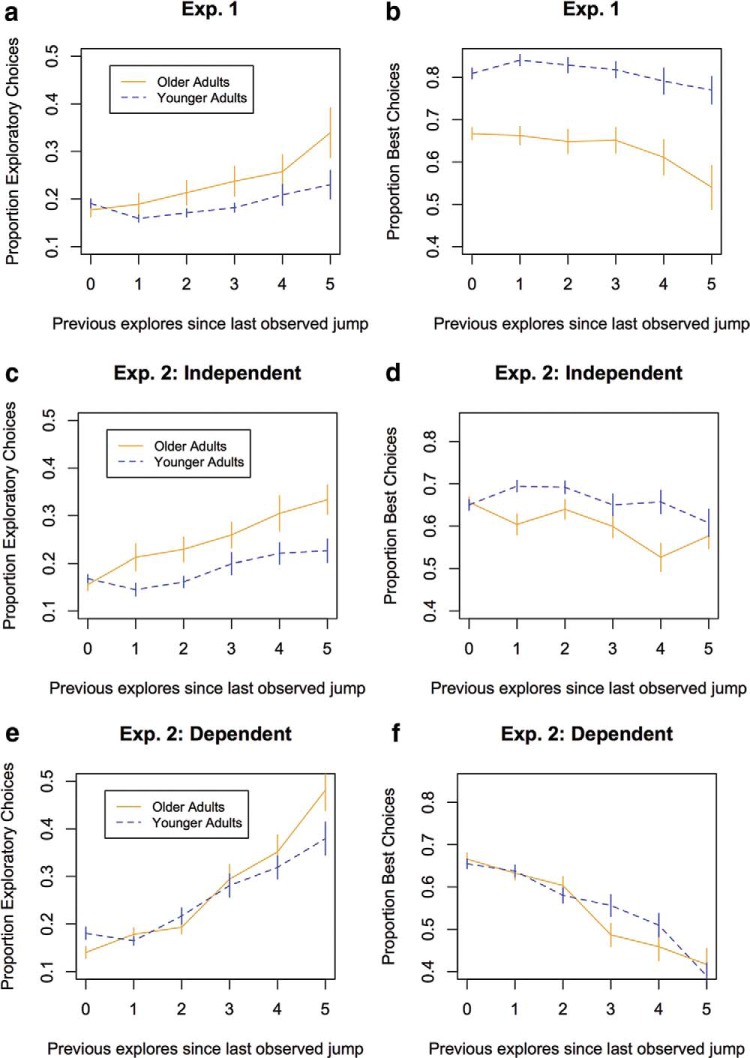
Knowledge effect analysis. Experiment 1 exploration rates as a function of the number of times the participant previously explored since the last time a jump was observed (a). An increasing pattern here indicates behavior similar to the gambler’s fallacy—acting as though a jump becomes more likely the longer it has been since the last jump occurred. Younger adults exhibited a relatively flat pattern while older adults produced a reliably increasing pattern, suggesting that older adults often assumed an incorrect mental representation of the environment. Experiment 1 performance as a function of the number of previously explored choices since the last observed jump (b). Performance decreases for both groups, but particularly for older adults, as exploration rates increase. The independent condition in Experiment 2 shows a similar pattern of results as Experiment 1, though with a smaller difference between groups in performance (c, d). In the dependent condition in Experiment 2, where jumps *do* become more likely the longer it has been since the last jump, both groups appropriately produce an increasing pattern in exploration rates (e, f). Performance shows a decreasing pattern. Error bars reflect standard errors. See the online article for the color version of this figure.

We also examined exploration rates as a function of the number of trials since a jump in reward values was observed (after controlling for the number of trials since the previous explore choice—i.e., the hazard rates) (see Figure 4a). This measure was designed to determine whether participants seemed to expect positive or negative autocorrelations in the environment. Specifically, we performed a mixed-effects logistic regression predicting exploratory choices from the number of trials since the last observed jump, age group, and their interaction, with participant as a random effect. Number of trials since the last exploratory choice and the Age Group × Trials Since the Last Observed Jump interaction were also entered into the regression to control for the hazard rates. Crucially, we found an Age Group × Trials Since the Last Observed Jump interaction (*z* = 2.68, *p* = .007), suggesting that the two groups differed on this measure. We then looked within each group to determine the nature of the interaction. There was a significant effect of the number of previous trials since the last observed jump on exploration rates for older adults (*z* = 3.429, *p* < .001, odds ratio [*OR*] = 1.008), but not for younger adults (*z* = −0.482, *p* = .630, *OR* = 0.999). This effect suggested that older adults expect a jump to become more likely over time even when the environment was recently sampled (i.e., they expected negative autocorrelation in trial outcomes). In other words, they acted as if the probability of a jump occurring depended on the outcome of previous trials, while the actual probability of a jump was constant and trials were sequentially independent.[Fig-anchor fig4]

We also examined performance as a function of the number of trials since the last observed jump (while controlling for the number of trials since the last explore choice) in order to determine whether incorrectly expecting negative autocorrelation in trial outcomes was hurting performance (see Figure 4b). A mixed-effects logistic regression predicting performance from the number of trials since the last observed jump, age group, and their interaction (while controlling for the number of trials since the last exploratory choice and the Age Group × Trials Since the Last Observed Jump interaction) was performed with participant as a random effect. Again, we found an Age Group × Trials Since the Last Observed Jump interaction on performance (*z* = −7.485, *p* < .001). Looking within each age group, we found that the number of trials since the last observed jump had a significant negative effect on performance for both older adults (*z* = −13.077, *p* < .001, *OR* = 0.975) and younger adults (*z* = −3.809, *p* < .001, *OR* = 0.994), though the coefficient was much larger for older adults than younger adults (−0.024 vs. −0.006). Both groups performed worse as more trials pass since the last observed jump, but older adults decreased at a faster rate, which could be due to their increased levels of exploration.

#### Neuropsychological test measures

As an exploratory analysis, we assessed the extent to which executive function influenced participants’ performance and strategies in the leapfrog task. A capacity limitation account of our results would predict that higher executive function scores would correlate positively with task performance. We conducted separate logistic regressions with each score on the neuropsychological tests related to executive function (i.e., TMT, FAS, and WCST) as a predictor of leapfrog task performance and of which model best characterized participants’ hazard rates. Surprisingly, task performance was not significantly predicted by any of the neuropsychological test scores, suggesting that differences in executive function do not explain differing task performance among our older adult participants. WCST Perseverative Error score was negatively related to being best characterized by the reflective linear model (*z* = −2.018, *p* = .044). No other neuropsychological test scores were related to the model fits.

### Discussion

Experiment 1 investigated exploratory decision-making in older and younger adults using a task designed to investigate exploratory behavior that is ostensibly not highly reliant on prior knowledge. In terms of task performance, our analyses revealed some reliable differences: on average, older participants responded more slowly than younger participants, and they made fewer optimal choices on the task. We did not find a difference in raw exploration rates as a function of age group, suggesting that the key difference between the two groups would be best characterized in terms of differences in strategy, rather than one group over- or underexploring. The highly similar rate of exploratory choices exhibited by both groups, despite the overall differences in performance, highlights the importance of investigating qualitative differences in strategy use between the groups.

Our analyses of participants’ choice strategies provided some evidence against a strict capacity limitation account of the age-related performance difference in this task. Younger adults were more often characterized by the linear model that represents good reflective strategy use compared with older adults, but most participants in both groups were more often best characterized by that model. Our analysis indicating that older adults expected negative autocorrelation in the trial outcomes (similar to the gambler’s fallacy) suggested that older adults may be using an alternative reflective strategy based on a different mental representation of the task. It is important to note that, although this strategy is suboptimal in this specific task, it is relatively demanding of cognitive processing resources. Indeed, this strategy may be even more memory demanding than the optimal one for this task because, under this strategy, uncertainty about task state does not return to baseline levels following an exploratory choice, and it thus requires that probabilities be tracked over longer periods of time. Additionally, the finding that the neuropsychological test measures relating to executive function did not positively relate to performance supported a knowledge effect rather than capacity limitation account of the performance difference.

## Experiment 2

Experiment 1 provided preliminary evidence supporting a knowledge effect account of the age-related performance differences in our task, while finding little evidence supporting a capacity limitation account. Experiment 2 directly tested an important implication of the knowledge effect interpretation, namely, that the relative performance of older adults to younger adults should depend on the underlying structure of the environment. The knowledge effect account would predict that older adults would do as well, or perhaps better, than younger adults in an environment that matches their expectations. In Experiment 2, we investigated this prediction by manipulating the task environment. In one condition (the dependent condition), trials were *not* independent, but instead the probability of a jump steadily increased over time until the next jump occurred. The other condition (the independent condition) replicated the structure of the standard leapfrog task used in Experiment 1, where each trial was sequentially independent.

A knowledge account predicts an interaction in performance such that younger adults will outperform older adults in the independent condition (as in Experiment 1), but not in the dependent condition. A capacity limitation account predicts that younger should perform better in both conditions.

### Method

#### Participants

To recruit a large and diverse sample of older and younger adults for Experiments 2, we used Amazon’s Mechanical Turk (http://www.mturk.com). Mechanical Turk has become increasingly popular among psychology researchers, and studies have validated it as a reliable methodology for collecting data in cognitive studies (Crump, McDonnell, & Gureckis, 2013). Participants over 60 years of age were recruited for our older adult sample, and participants between 18 and 30 years of age were recruited for our younger adult sample. Potential participants between the ages of 30 and 60 years were informed that they did not qualify for participate.

One hundred thirty-nine younger and 137 older adults participated in the experiment in one of two conditions (see Procedure section). There were 68 older adults and 70 younger adults in the independent condition and 69 older adults and 69 younger adults in the dependent condition. Younger adults’ mean age was 22.46 years (range: 18−30 years) in the independent condition and 23.45 years (range: 19−30 years) in the dependent condition. Older adults’ mean age was 64.09 years (range: 60−75 years) in the independent condition and 64.72 years (range: 60−77 years) in the dependent condition.

#### Procedure

The task procedure was similar to that used in Experiment 1, with two main differences. First, participants completed the task remotely through Amazon Mechanical Turk rather than in the lab. PsiTurk (http//www.psiTurk.org; McDonnell et al., 2012) was used to develop the experiment for use on Mechanical Turk and as an interface for interaction with the Mechanical Turk system. Second, the experiment included two conditions that differed in the underlying probabilities that determine when reward jumps occur. In the independent condition, the probability of a jump on a particular trial was independent of the outcome of other trials, set at a constant 0.075 probability. This condition was a procedural replication of Experiment 1. In the dependent condition, the probability of a jump started at 0.01 and increased linearly by 0.01 on each trial until a jump occurred. When a jump occurred, the probability reset to 0.01 for the next trial.

As in Experiment 1, after receiving instructions, participants passively viewed 200 training trials to acclimate to the rate of change in the reward values. Each training trial lasted for 0.5 s, immediately followed by the next trial. The current trial number was displayed at the center of the screen at all times. Reward values for the options were not shown. Instead, jumps were indicated by a yellow box highlighting the option that changed. At the end of each block of 100 training trials, participants estimated the number of jumps they had expected on the next block.

Following training, participants completed 200 trials of the main task. On each trial, the word CHOOSE appeared on the screen, and participants chose one of the two options using the keyboard. The chosen option was highlighted and the reward received for the choice was then presented in the center of the screen for 1.5 s. Immediately after reward presentation, the next trial began. Instructions did not differ between conditions and were the same as used in Experiment 1.

### Results

#### Performance, RTs, and exploration rates

The key measure of Experiment 2 was a 2 × 2 (Age Group × Condition) analysis of variance (ANOVA) on task performance since a knowledge effect and a capacity limitation account produce different predictions. This test revealed a significant interaction, *F*(1, 272) = 4.447, *p* = .036, and a main effect of condition, *F*(1, 272) = 5.719, *p* = .018. Younger adults scored higher than older adults (0.667 vs. 0.638) on the independent condition, *t*(136) = 2.086, *p* = .039, *d* = 0.356, but not on the dependent condition, (0.624 vs. 0.635), *t*(136) = 0.856, *p* = .394, *d* = 0.146 (see Figure 2c). This result was consistent with the prediction of a knowledge effect.

A 2 × 2 (Age Group × Condition) ANOVA on median RT revealed only a main effect of age group, *F*(1, 272) = 128.813, *p* < .001. Younger adults responded more quickly in both the independent condition (201 ms vs. 357 ms), *t*(136) = 8.862, *p* < .001, *d* = 1.509, and the dependent condition (235 ms vs. 370 ms), *t*(136) = 7.227, *p* < .001, *d* = 1.231 (see Figure 2d). A 2 × 2 (Age Group × Condition) ANOVA on exploration rate did not show significant main effects of age group, *F*(1, 272) = 0.100, *p* = .752, or condition, *F*(1, 272) = 0.700, *p* = .403, or a significant interaction, *F*(1, 272) = 2.093, *p* = .149. Exploration rates did not differ between younger and older adults for either the independent condition (0.144 vs. 0.156), *t*(136) = 0.812, *p* = .418, *d* = 0.138, or the dependent condition (0.168 vs. 0.149), *t*(136) = 1.226, *p* = .222, *d* = 0.209.

#### Autocorrelation analysis

As in Experiment 1, we investigated whether participants’ behavior was consistent with expecting positive or negative autocorrelations in the environment. We examined exploration rates as a function of the number of trials since a jump in reward values was observed (see Figures 4c−4f). We first looked across both conditions. Mixed-effects logistic regressions were performed predicting exploratory choices and performance from the number of trials since the last observed jump, condition, age group, and their interactions (while controlling for the number of trials since the last exploratory choice and its interactions with group and condition), with participant as a random effect. The three-way (Condition × Age Group × Trials Since Last Observed Jump) interaction was not significant for exploration rate (*z* = −0.42, *p* = .678). The regression on performance did reveal a significant three-way interaction, though (*z* = 3.14, *p* = .002).

We then looked within the independent condition to see whether the effect from Experiment 1 replicated in this population. We performed a mixed-effects logistic regression predicting exploratory choices from the number of trials since the last observed jump, age group, and their interaction (while controlling for the number of trials since the last exploratory choice and its interaction with age group), with participant as a random effect (see Figure 4c). We found a marginal Age Group × Trials Since the Last Observed Jump interaction (*z* = 1.774, *p* = .076). The qualitative pattern from Experiment 1 was replicated, but was not statistically significant. The effect was not statistically significant within either group (*z* = 1.505, *p* = .132, *OR* = 1.003, for older adults; *z* = −0.715, *p* = .475, *OR* = 0.999, for younger adults).

We also looked at performance for the independent condition (see Figure 4d). We performed a mixed-effects logistic regression predicting performance from the number of trials since the last observed jump, age group, and their interaction (while controlling for the number of trials since the last exploratory choice and its interaction with age group), with participant as a random effect. There was a significant Age Group × Trials Since the Last Observed Jump interaction (*z* = −5.464, *p* < .001). Both older adults (*z* = −16.698, *p* < .001, *OR* = 0.974) and younger adults (*z* = −10.15, *p* < .001, *OR* = 0.985) had significant negative relationships between performance and the number of trials since the last observed jump, with a stronger effect for older adults than younger adults.

In the dependent condition, the environment contained negative autocorrelation, and both groups seem to have picked up on it. The Age Group × Trials Since the Last Observed Jump interaction on exploration rate was not significant (*z* = 0.854, *p* = .393), but there was a significant main effect of trials since the last observed jump (*z* = 7.121, *p* < .001, *OR* = 1.024; see Figure 4e). This effect was also statistically significant in both the older adult group (*z* = 10.04, *p* < .001, *OR* = 1.034) and the younger adult group (*z* = 7.661, *p* < .001, *OR* = 1.026). Similarly, for performance, the interaction was not significant (*z* = 0.51, *p* = .612), but the main effect of trials since last observed jump was significant (*z* = −29.55, *p* < .001, *OR* = 0.923; see Figure 4f). Again, this effect occurred in both groups (older adults: *z* = −30.49, *p* < .001, *OR* = 0.923; younger adults: *z* = −29.46, *p* < .001, *OR* = 0.922). Overall, the results of Experiment 2 confirmed the central predictions of the knowledge account—that older and younger adults would not differ in performance when trial outcomes were negatively autocorrelated, but that older adults would perform worse when outcomes were sequentially independent (replicating the results of Experiment 1).

## General Discussion

We investigated exploratory decision-making in older and younger adults to determine the extent to which age-related performance differences reflect either knowledge effects or capacity limitations due to cognitive decline. Cognitive decline is often invoked as an explanation for age-related decision-making differences, whereas explanations based on the additional knowledge and experience that older adults are likely to have acquired are less often considered. A capacity limitation account predicts that older adults should underperform and rely on less cognitively demanding strategies than younger adults across the board. An account based on knowledge effects predicts that whether older adults perform equivalent to, worse than, or better than younger adults depends on the nature of the task. Although these viewpoints are not exclusive, we suspect that evidence is routinely interpreted as favoring the capacity limitation interpretation when it may better be explained in terms of increased knowledge and experience.

Experiment 1 provided some initial support for a knowledge-related interpretation of the performance difference between the older and younger adults. Analyses directed toward examining the timing of exploratory choices revealed that younger adults used more effective strategies for the current task, but both groups showed signs of maintaining rich representations of the environment to track probabilities over time in a reflective manner. Older adults used a cognitively demanding reflective strategy, just not the optimal one for that decision environment. This is consistent with other recent work that has shown older and younger adults apply qualitatively different strategies in cognitive tasks (e.g., Vaportzis, Georgiou-Karistianis, & Stout, 2013). Older adults seemed to expect a negative autocorrelation in the probability of a jump occurring—that a jump would be more likely the longer it had been since the previous jump—while younger adults did not. Importantly, whether or not this type of behavior is suboptimal depends on the underlying structure of the environment. This type of reasoning is effective in many real-world decision-making scenarios, and we speculate that the tendency to expect this type of structure in the environment may result from real-world experience.

Experiment 2 provided further support for a knowledge-based interpretation by testing one of its key interpretations—that the underlying structure of the environment is critical in determining the relative performance of older adults to younger adults. We found that when trials were not sequentially independent—the probability of a jump instead increasing over time—older adults performed as well as younger adults. It is perhaps particularly surprising that older adults performed as well as younger adults in the dependent condition, but not the independent condition, because the optimal strategy for the dependent condition required tracking probabilities over a longer period of time, making good performance *more* dependent on processing capacity.

### Experience, Processing, and Age

Taken together, the results of these two experiments indicate that the different reflective strategies employed by older and younger adults account for some of the differences in performance observed, and that these differences are not simply indicative of diminished cognitive capacities. It is perhaps worth reflecting on the fact that, without our analyses investigating participants’ strategies, we would likely have interpreted the lower performance of the older adults as further evidence of their diminished cognitive capacities, and taken our results to have shown that people’s ability to engage in reflective decision-making declines with age.

In doing so, we might have cited the work showing that attention, working memory, and executive control “decline” with age (e.g., Salthouse, 2004, 2009). We could have noted that normal dopamine function in the striatum, a critical component of learning and decision-making, declines with age, and there is a reduced striatal and frontostriatal representation of reward prediction errors in older adults (e.g., Eppinger, Schuck, et al., 2013; Samanez-Larkin, Worthy, Mata, McClure, & Knutson, 2014). We could have observed that this may be related to compromised white matter integrity in frontostriatal circuits (e.g., Samanez-Larkin, Levens, Perry, Dougherty, & Knutson, 2012). And we might have noted work that has shown older adults prefer fewer choice options than younger adults (e.g., Reed, Mikels, & Simon, 2008), which is consistent with a reduced ability to maintain and process information related to a larger number of options, and which would have been consistent with older adults’ “deficient capacities for reflective decision making.”

The question still remains why older adults are relying on one particular strategy, even though it leads to suboptimal performance in some scenarios. One hypothesis is that older adults have a lower ability to flexibly adapt their strategy to the task at hand. This hypothesis is consistent with studies that have found performance deficits in task switching for older adults (Cepeda, Kramer, & Gonzalez de Sather, 2001; Gold, Powell, Xuan, Jicha, & Smith, 2010; Kray & Lindenberger, 2000). One possibility is that the beliefs about the environment that older adults seem to display (i.e., expecting negative autocorrelation similar to the gambler’s fallacy) represent a default expectation, and younger adults are better at modifying their behavior when those expectations are defied—as in the independent condition.

A related, yet slightly different hypothesis is that exploratory decision-making in older adults may have been tuned by the wealth of experience making decisions in the real world—where probabilities *do* often depend on past outcomes and individual events are rarely independent. Increased experience could make older adults more likely to apply a strategy that may often be effective in real-world exploratory decision-making scenarios, but which fails in the relatively artificial environment of the standard (sequentially independent) leapfrog task. This idea is consistent with rational accounts of cognition (Anderson, 1990; Fawcett et al., 2014), which propose that cognitive systems are optimized for the statistical structure of the environment in which they operate. We speculate that, when estimating probabilities in the real world, the gambler’s fallacy is often an effective assumption. Behavior consistent with the gambler’s fallacy is reasonable in many scenarios where individual events are not sequentially independent, where the probability of a change does increase with time.

It should be noted that this hypothesis is somewhat speculative. Real-world decision-making encompasses a large number of different decision environments with varying underlying probability structures. While we believe that scenarios with negative autocorrelation in event probabilities over time (i.e., where the gambler’s fallacy is not fallacious) are particularly common, we have little concrete evidence to support this claim, and it is likely impossible to enumerate the various decision scenarios that a person encounters and to compare the frequency of different underlying probability structures. Second, this study provided no direct evidence that experience caused the differences in strategy use between the two age groups.

A major limitation of this study is its correlational, as opposed to longitudinal, nature. As with all correlational studies of aging, it is impossible to distinguish effects of cohort from true aging effects. Determining the extent to which experience influences beliefs and strategies underlying decision-making, and how it contributes to age-related differences in cognition, is an important topic for future research. Another limitation of the current study is that we did not collect neuropsychological test measures on our younger adult sample in Experiment 1, making it difficult to compare the two age groups on general cognitive function or to assess its effect on task performance.

What is interesting is that, even though we tried to model the processes that appeared to be subject to age-related change, processes usually are either not formally modeled at all or, where models are offered, they usually failed to acknowledge or control for potential knowledge or experience effects. Seeking to better formally model the effects of experience on task and response representations represents an important line of future research for cognitive aging research. Experience effects may pervade many domains of cognition studied in aging, and it is crucial that we identify and understand these effects if we are to comprehend the influence of normal aging on cognition.

### Conclusion

Decision-making is a cognitive skill that is critical throughout life, so it is important to understand how it changes over the life span. Exploratory behavior is a key aspect of decision-making that can give us insight into how decision-making changes with aging. We found that older and younger adults engage in qualitatively different exploration strategies. These differences led to worse performance for older adults than for younger adults in one decision environment, but not another, more complex environment. Our results highlight the importance of considering potential knowledge effects, which may often be misinterpreted as further evidence of capacity limitations due to neural decline, when investigating age-related differences in cognition. Our task is simpler than many real-life decision-making scenarios, where the decision environment is much more complex, and so age-related differences in exploratory strategy use may manifestly differ outside of the laboratory. Due to a lifetime of experience making decisions, older adults may be able to rely on well-learned strategies to make effective decisions in the real world.

## Figures and Tables

**Table 1 tbl1:** Neuropsychological Test Z Scores

Variables	*M* (*SD*)	Range
Neuropsychological test		
Digit Span	0.487 (0.971)	−1.00 to 3.00
CVLT Delayed Recall (Free)	0.509 (1.01)	−1.50 to 2.50
CVLT Immediate Recall (Free)	0.625 (0.949)	−1.50 to 2.50
CVLT Delayed Recall (Cued)	0.308 (0.986)	−2.50 to 2.00
CVLT Immediate Recall (Cued)	0.442 (0.978)	−1.50 to 2.50
CVLT Recognition False Positives	−0.029 (1.29)	−1.00 to 4.50
CVLT Recognition True Positives	−0.058 (1.02)	−4.00 to 1.00
FAS	−0.029 (0.965)	−2.56 to 2.48
Trail-Making Test Part A	−0.483 (0.764)	−1.42 to 1.95
Trail-Making Test Part B	−0.544 (0.506)	−2.07 to 1.01
WCST Errors	0.408 (0.921)	−1.50 to 2.50
WCST Perseverative Errors	0.667 (0.851)	−0.80 to 3.00
Demographic information		
Age (years)	67.02 (5.13)	60 to 88
Years of education	16.60 (2.75)	10 to 25
*Note*. CVLT = California Verbal Learning Test; WCST = Wisconsin Card Sorting Task.		

**Figure B1 fig5:**
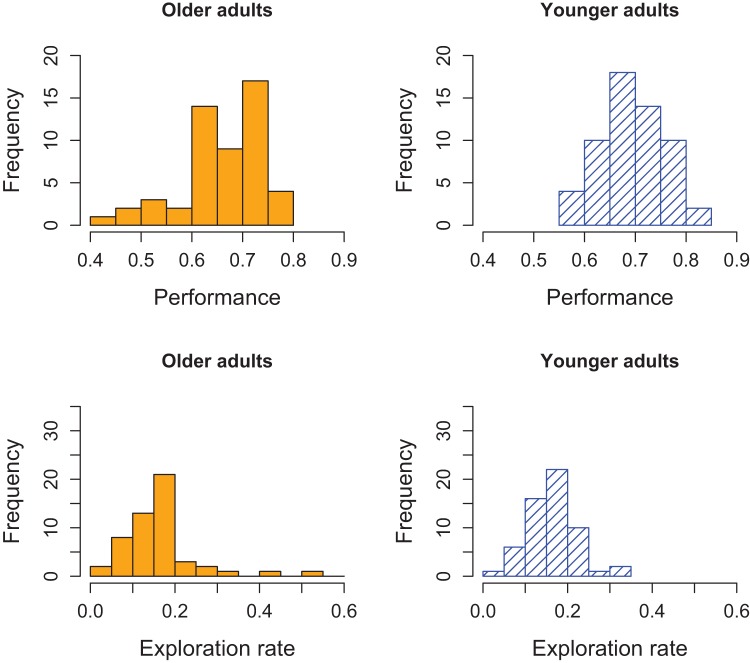
Histograms of performance and exploration rates for older and younger adults in Experiment 1. See the online article for the color version of this figure.

## A Models Used to Generate Reflective and Reflexive Predictions

To demonstrate the key patterns of behavior that characterize reflexive and good reflective strategies in the leapfrog task, we ran simulations of the ideal reflective and reflexive models. These predictions are discussed in the Introduction, and the key pattern of results is plotted above (see Figure 1c).

The best reflective strategy for this task is an ideal actor that produces the optimal performance in the task, exploring when it is most valuable (i.e., when there is more uncertainty) in order to optimize long-term rewards. Optimal choices for the leapfrog task used by this model are computed by specifying the task as a partially observable Markov decision process (Kaelbling, Littman, & Cassandra, 1998).

The best reflexive model in this task is a standard reinforcement learning model with a learning rate of one. Exploration is a result of a stochastic decision process, using a Softmax choice rule (Sutton & Barto, 1998). The Softmax inverse temperature parameter was set to the optimal value for the model, as determined from simulations of the task at various parameter settings. This model exploits with equal probability on each trial. For full detailed descriptions of these models, see Knox et al. (2012).

## B Distribution of Performance and Exploration Rate

Because individuals vary in terms of strategies in the leapfrog task, and different strategies may lead to qualitatively different patterns of performance, we examined the distributions of two key measures (performance and exploration rate) within each group to determine whether the mean statistics compared in the Results section of Experiment 1 truly reflect the majority of individual participants (see Figure B1). The distributions of both measures in each group are clearly unimodal and centered on the mean value.[Fig-anchor fig5]

## References

[c1] Alloy L. B., & Tabachnik N. (1984). Assessment of covariation by humans and animals: The joint influence of prior expectations and current situational information. Psychological Review, 91, 112–149. 10.1037/0033-295X.91.1.112 6571422

[c2] Anderson J. R. (1990). The adaptive character of thought. Hillsdale, NJ: Lawrence Erlbaum Associates.

[c3] Badre D., Doll B. B., Long N. M., & Frank M. J. (2012). Rostrolateral prefrontal cortex and individual differences in uncertainty-driven exploration. Neuron, 73, 595–607. 10.1016/j.neuron.2011.12.025 22325209 PMC3285405

[c4] Barron G., & Leider S. (2010). The role of experience in the gambler’s fallacy. Journal of Behavioral Decision Making, 23, 117–129. 10.1002/bdm.676

[c5] Blanchard T. C., Wilke A., & Hayden B. Y. (2014). Hot-hand bias in rhesus monkeys. Journal of Experimental Psychology: Animal Learning and Cognition, 40, 280–286. 10.1037/xan0000033 25545977

[c6] Blanco N. J., Love B. C., Cooper J. A., McGeary J. E., Knopik V. S., & Maddox W. T. (2015). A frontal dopamine system for reflective exploratory behavior. Neurobiology of Learning and Memory, 123, 84–91. 10.1016/j.nlm.2015.05.004 26004676 PMC4530051

[c7] Blanco N. J., Otto A. R., Maddox W. T., Beevers C. G., & Love B. C. (2013). The influence of depression symptoms on exploratory decision-making. Cognition, 129, 563–568. 10.1016/j.cognition.2013.08.018 24055832 PMC3809321

[c8] Burke S. N., & Barnes C. A. (2006). Neural plasticity in the ageing brain. Nature Reviews Neuroscience, 7, 30–40. 10.1038/nrn1809 16371948

[c9] Castel A. D., Rossi A. D., & McGillivray S. (2012). Beliefs about the “hot hand” in basketball across the adult life span. Psychology and Aging, 27, 601–605. 10.1037/a0026991 22288426

[c10] Cepeda N. J., Kramer A. F., & Gonzalez de Sather J. C. (2001). Changes in executive control across the life span: Examination of task-switching performance. Developmental Psychology, 37, 715–730. 10.1037/0012-1649.37.5.715 11552766

[c11] Chowdhury R., Guitart-Masip M., Lambert C., Dayan P., Huys Q., Düzel E., & Dolan R. J. (2013). Dopamine restores reward prediction errors in old age. Nature Neuroscience, 16, 648–653. 10.1038/nn.3364 23525044 PMC3672991

[c12] Cooper J. A., Worthy D. A., Gorlick M. A., & Maddox W. T. (2013). Scaffolding across the lifespan in history-dependent decision-making. Psychology and Aging, 28, 505–514. 10.1037/a0032717 23795765 PMC3694731

[c13] Crump M. J., McDonnell J. V., & Gureckis T. M. (2013). Evaluating Amazon’s Mechanical Turk as a tool for experimental behavioral research. PLoS ONE, 8, 10.1371/journal.pone.0057410 PMC3596391 23516406

[c14] Daw N. D., Niv Y., & Dayan P. (2005). Uncertainty-based competition between prefrontal and dorsolateral striatal systems for behavioral control. Nature Neuroscience, 8, 1704–1711. 10.1038/nn1560 16286932

[c15] Denburg N. L., Tranel D., & Bechara A. (2005). The ability to decide advantageously declines prematurely in some normal older persons. Neuropsychologia, 43, 1099–1106. 10.1016/j.neuropsychologia.2004.09.012 15769495

[c16] Eppinger B., Schuck N. W., Nystrom L. E., & Cohen J. D. (2013). Reduced striatal responses to reward prediction errors in older compared with younger adults. The Journal of Neuroscience, 33, 9905–9912. 10.1523/JNEUROSCI.2942-12.2013 23761885 PMC3682384

[c17] Eppinger B., Walter M., Heekeren H. R., & Li S. C. (2013). Of goals and habits: Age-related and individual differences in goal-directed decision-making. Frontiers in Neuroscience, 7, 253 10.3389/fnins.2013.00253 24399925 PMC3871973

[c18] Falk R., & Konold C. (1997). Making sense of randomness: Implicit encoding as a basis for judgment. Psychological Review, 104, 301–318. 10.1037/0033-295X.104.2.301

[c19] Fawcett T. W., Fallenstein B., Higginson A. D., Houston A. I., Mallpress D. E., Trimmer P. C., . . . Modelling Animal Decisions Group (2014). The evolution of decision rules in complex environments. Trends in Cognitive Sciences, 18, 153–161. 24467913 10.1016/j.tics.2013.12.012

[c20] Fridlund A., & Delis D. C. (1987). CVLT research edition: Administration and scoring software. New York, NY: The Psychological Corporation.

[c21] Gold B. T., Powell D. K., Xuan L., Jicha G. A., & Smith C. D. (2010). Age-related slowing of task switching is associated with decreased integrity of frontoparietal white matter. Neurobiology of Aging, 31, 512–522. 10.1016/j.neurobiolaging.2008.04.005 18495298 PMC2815097

[c22] Hahn U., & Warren P. A. (2009). Perceptions of randomness: Why three heads are better than four. Psychological Review, 116, 454–461. 10.1037/a0015241 19348550

[c23] Hahn U., & Warren P. A. (2010). Why three heads are a better bet than four: A reply to Sun, Tweney and Wang. Psychological Review, 117, 706–711. 10.1037/a0019037 20438245

[c24] Head D., Kennedy K. M., Rodrigue K. M., & Raz N. (2009). Age differences in perseveration: Cognitive and neuroanatomical mediators of performance on the Wisconsin Card Sorting Test. Neuropsychologia, 47, 1200–1203. 10.1016/j.neuropsychologia.2009.01.003 19166863 PMC2649973

[c25] Heaton R. K. (1981). A manual for the Wisconsin Card Sorting Test. Odessa, FL: Psychological Assessment Resources.

[c26] Hills T. T., Mata R., Wilke A., & Samanez-Larkin G. R. (2013). Mechanisms of age-related decline in memory search across the adult life span. Developmental Psychology, 49, 2396–2404. 10.1037/a0032272 23586941 PMC3842414

[c27] Kaelbling L. P., Littman M. L., & Cassandra A. R. (1998). Planning and acting in partially observable stochastic domains. Artificial Intelligence, 101, 99–134. 10.1016/S0004-3702(98)00023-X

[c28] Knox W. B., Otto A. R., Stone P., & Love B. C. (2012). The nature of belief-directed exploratory choice in human decision-making. Frontiers in Psychology, 2, 398 10.3389/fpsyg.2011.00398 22319503 PMC3269072

[c29] Kray J., & Lindenberger U. (2000). Adult age differences in task switching. Psychology and Aging, 15, 126–147. 10.1037/0882-7974.15.1.126 10755295

[c30] Lezak M. (1995). Neuropsychological testing (3rd ed.). New York, NY: Oxford University Press.

[c31] Li Y., Baldassi M., Johnson E. J., & Weber E. U. (2013). Complementary cognitive capabilities, economic decision making, and aging. Psychology and Aging, 28, 595–613. 10.1037/a0034172 24040999 PMC4086863

[c32] Mata R., & Nunes L. (2010). When less is enough: Cognitive aging, information search, and decision quality in consumer choice. Psychology and Aging, 25, 289–298. 10.1037/a0017927 20545414

[c33] McDonnell J. V., Martin J. B., Markant D. B., Coenen A., Rich A. S., & Gureckis T. M. (2012). psiTurk (Version 1. 02) [Software]. New York, NY: New York University Available from https://github.com/NYUCCL/psiTurk

[c34] Otto A. R., Knox W. B., Markman A. B., & Love B. C. (2014). Physiological and behavioral signatures of reflective exploratory choice. Cognitive, Affective & Behavioral Neuroscience, 14, 1167–1183. 10.3758/s13415-014-0260-4 24664860

[c35] Peisah C., Finkel S., Shulman K., Melding P., Luxenberg J., Heinik J., . . . International Psychogeriatric Association Task Force on Wills and Undue Influence (2009). The wills of older people: Risk factors for undue influence. International Psychogeriatrics, 21, 7–15. 10.1017/S1041610208008120 19040788

[c36] Ramscar M., Hendrix P., Love B., & Baayen H. (2013). Learning is not decline: The mental lexicon as a window into cognition across the lifespan. The Mental Lexicon, 8, 450–481. 10.1075/ml.8.3.08ram

[c37] Ramscar M., Hendrix P., Shaoul C., Milin P., & Baayen H. (2014). The myth of cognitive decline: Non-linear dynamics of lifelong learning. Topics in Cognitive Science, 6, 5–42. 10.1111/tops.12078 24421073

[c38] Reed A. E., Mikels J. A., & Simon K. I. (2008). Older adults prefer less choice than young adults. Psychology and Aging, 23, 671–675. 10.1037/a0012772 18808256 PMC2631411

[c39] Salthouse T. A. (2004). What and when of cognitive aging. Current Directions in Psychological Science, 13, 140–144. 10.1111/j.0963-7214.2004.00293.x PMC4219741 25382943

[c40] Salthouse T. A. (2009). When does age-related cognitive decline begin? Neurobiology of Aging, 30, 507–514. 10.1016/j.neurobiolaging.2008.09.023 19231028 PMC2683339

[c41] Samanez-Larkin G. R., Kuhnen C. M., Yoo D. J., & Knutson B. (2010). Variability in nucleus accumbens activity mediates age-related suboptimal financial risk taking. The Journal of Neuroscience, 30, 1426–1434. 10.1523/JNEUROSCI.4902-09.2010 20107069 PMC2821055

[c42] Samanez-Larkin G. R., Levens S. M., Perry L. M., Dougherty R. F., & Knutson B. (2012). Frontostriatal white matter integrity mediates adult age differences in probabilistic reward learning. The Journal of Neuroscience, 32, 5333–5337. 10.1523/JNEUROSCI.5756-11.2012 22496578 PMC3744863

[c43] Samanez-Larkin G. R., Worthy D. A., Mata R., McClure S. M., & Knutson B. (2014). Adult age differences in frontostriatal representation of prediction error but not reward outcome. Cognitive, Affective & Behavioral Neuroscience, 14, 672–682. 10.3758/s13415-014-0297-4 PMC4072917 24853269

[c44] Scheibehenne B., Wilke A., & Todd P. M. (2011). Expectations of clumpy resources influence predictions of sequential events. Evolution and Human Behavior, 32, 326–333. 10.1016/j.evolhumbehav.2010.11.003

[c45] Schwarz G. (1978). Estimating the dimension of a model. Annals of Statistics, 6, 461–464. 10.1214/aos/1176344136

[c46] Sutton R., & Barto A. G. (1998). Reinforcement learning. Cambridge, MA: MIT Press.

[c47] Vaportzis E., Georgiou-Karistianis N., & Stout J. C. (2013). Dual task performance in normal aging: A comparison of choice reaction time tasks. PLoS ONE, 8, 10.1371/journal.pone.0060265 PMC3605385 23555937

[c48] Wechsler D. (1997). Wechsler Adult Intelligence Scale: Administration and scoring manual (3rd ed.). San Antonio, TX: Psychological Corporation.

[c49] Worthy D. A., Cooper J. A., Byrne K. A., Gorlick M. A., & Maddox W. T. (2014). State-based versus reward-based motivation in younger and older adults. Cognitive, Affective & Behavioral Neuroscience, 14, 1208–1220. 10.3758/s13415-014-0293-8 PMC4221294 24845527

[c50] Worthy D. A., Gorlick M. A., Pacheco J. L., Schnyer D. M., & Maddox W. T. (2011). With age comes wisdom: Decision making in younger and older adults. Psychological Science, 22, 1375–1380. 10.1177/0956797611420301 21960248 PMC3212636

[c51] Worthy D. A., & Maddox W. T. (2012). Age-based differences in strategy use in choice tasks. Frontiers in Neuroscience, 5, 145 10.3389/fnins.2011.00145 22232573 PMC3252562

